# Comparing the structure and photoluminescence properties of Bi-doped lead-free double perovskites prepared by solution-based synthesis and green mechanochemistry

**DOI:** 10.1038/s41598-026-66090-3

**Published:** 2026-08-10

**Authors:** Shweta Chahal, Christian Würth, Arne Güttler, Ute Resch-Genger, Andrea Simone Stucchi de Camargo

**Affiliations:** 1 Federal Institute for Material Research and Testing (BAM), 12489 Berlin, Germany; 2 Otto-Schott Institute of Materials Research (OSIM), Friedrich-Schiller University, 07743 Jena, Germany

**Keywords:** Chemistry, Materials science, Nanoscience and technology, Physics

## Abstract

In search for less environmentally harmful materials, lead-free double halide perovskites (DPs) have emerged as promising candidates with tuneable photoluminescence properties. In this study, we present a comparative analysis of DPs with composition Cs_2_Ag_0.4_Na_0.6_Bi_y_In_1−y_Cl_6_ (CANBIC, y = 0.01–0.04, y is Bi content in mol%) obtained via solution-based (SB) and green solvent-free mechanochemical (MC) methods. Comprehensive characterization of the resulting materials was performed via X-ray diffraction (XRD), thermogravimetric analysis (TGA), scanning and transmission electron microscopy (SEM/TEM), optical diffuse reflectance and photoluminescence (PL) spectroscopies. Both synthesis routes yielded cubic (Fm-3 m) perovskite structures, with all CANBIC samples exhibiting broad emission spectra ranging from 400 to 900 nm (1.3–3.1 eV), originating from the recombination of self-trapped excitons (STEs). Bi^3+^ doping significantly enhanced photoluminescence quantum yield (PLQY) up to 84%. The solution-based (SB) method produced samples with higher PLQY, attributed to improved control over particle morphology and dopant distribution as compared to the mechanochemistry (MC) route. Thermal analysis confirmed the structural integrity of CANBIC samples up to 300 °C, with no significant decomposition below ~ 560 °C, indicating their suitability for optoelectronic applications requiring moderate thermal stability. This work provides valuable insights into the relationship between synthesis methodology and material performance, advancing the development of environmentally friendly lead-free perovskites for next-generation optoelectronic devices such as white-LEDs.

**Supplementary Information:**

The online version contains supplementary material available at 10.1038/s41598-026-66090-3.

## Introduction

White light-emitting diodes (WLEDs) are presently in high demand for applications in the automotive, biomedical, and display technology fields, due to their superior energy efficiency, long operational lifetimes, and higher reliability compared to conventional lighting systems. Pb-based halide perovskites have demonstrated exceptional optoelectronic properties, including high absorption coefficients, elevated charge carrier mobilities, and high photoluminescence yield^1–4^. However, the inherent toxicity and environmental instability of lead compounds, particularly their high solubility in water, impose substantial risks to ecosystems and human health. These concerns have prompted the urgent search for Pb-free alternatives with similar or superior optoelectronic characteristics such as Sn^2+^ and Ge^2+^-based perovskite quantum dots^5^. Nevertheless, their susceptibility to oxidation under ambient conditions compromises their structural integrity and long-term stability. To address these limitations, cation substitution strategies have been explored, including the incorporation of monovalent, trivalent, and tetravalent cations. A particularly promising material class is the Pb-free double halide perovskites (DPs), with general formula A_2_B^+^B^3+^X_6_ (A = Cs^+^, Rb^+^; B^+^ = Na^+^, K^+^, Cu^+^, Ag^+^; B^3+^ = In^3+^, Sb^3+^, Bi^3+^)^6^. These compounds exhibit either direct or indirect bandgap characteristics. For example, Cs_2_AgInCl_6_ possesses a direct bandgap of 3.51 eV, and optical transitions from the valence band maximum (VBM), composed of In 4d/Ag 4d and Cl 3p orbitals, to the conduction band minimum (CBM), primarily formed by delocalized In 5s/Ag 5s states are parity forbidden. However, this transition is parity-forbidden due to the even symmetry of both band-edge states, resulting in extremely weak emission, while Cs_2_B^+^BiX_6_ and CsB^+^SbX_6_ typically display indirect bandgaps. Thus, despite these promising characteristics, the inherent parity-forbidden transitions due to inversion symmetry in these materials result in the formation of dark self-trapped excitons (STEs), which significantly reduce the photoluminescence quantum yield (PLQY)^7^. To circumvent these limitations, doping strategies have been employed with Mn^2+^, for example, which shifts the PL emission of Cs_2_AgInCl_6_ from 560 nm to 620 nm and enhances PLQY from 1.6% to 16%, due to the introduction of Mn^2+^ d–d transitions.

The optoelectronic properties of double perovskites depend on their band gap and electronic structure, determined by the atomic orbitals and occupancy of the B^+^ and B^3+^ sites^8^. This fundamental selection rule leads to a low radiative recombination efficiency and results in an optical bandgap exceeding the electronic bandgap. Co-doping with Na^+^ and Bi^3+^ has also been shown to modulate the wavefunction symmetry of STEs and further improve PLQY. This introduces 6s^2^ lone pair states that can mediate parity-allowed transitions, thereby enhancing light absorption and reducing the effective optical bandgap^9^.

Data reported for similar Bi^3+^- doped Cs_2_AgInCl_6_ systems provide detailed electronic band structures. Bi^3+^ introduces occupied 6s^2^ lone-pair states situated energetically above the valence band maximum (VBM), which hybridize with Cl 3p orbitals and shift the VBM to the higher energy level, narrowing the optical bandgap^10^. Simultaneously, Bi 6p states can partially relieve the parity-forbidden character of the band edge transition and activate STE-mediated radiative recombination^11^.

Despite the growing interest in these lead-free double perovskites (DPs) compositions, achieving phase-purity and high crystallinity requires precise control over reaction kinetics, precursor interactions, and crystallization pathways, which can be challenging^12^. Most of the synthesis routes yielding high-quality DPs require substantial energy input, typically involving hydrothermal treatments or high-temperature calcination. Also, hazardous reagents such as hydrochloric acid for chloride-based DPs and unstable or corrosive precursors for bromide and iodide analogues are employed, imposing environmental and safety concerns. Therefore, to enable sustainable and scalable fabrication under ambient conditions, alternative synthesis strategies are sought, including low-temperature, saturation-driven, and antisolvent precipitation methods. Still, these methods often result in poorly controlled phase formation, yielding mixtures of perovskite and non-perovskite phases.

Herein, we investigate two ambient-condition synthesis routes that eliminate the requirement for thermal activation and hazardous chemicals: solution-base (SB) and mechanochemical (MC) methods. The SB approach involves controlled precursor concentrations and solvent evaporation to induce crystallization, while MC synthesis utilizes mechanical energy to drive rapid, solvent-free reactions. MC offers several advantages over SB, including shorter reaction times, absence of solvent, and suppression of secondary phase formation. It has been successfully applied to the synthesis of halide DPs such as Cs_2_AgBiX_6_ (X = Cl, Br, I) and Cs_2_NaBiCl_6_ under mild, ambient conditions and can yield phase-pure Cs_2_AgBiBr_6_^13,14^. Also, a closely related study by Huang et al.^15^ on the synthesis of Bi^3+^-doped Cs_2_Ag_0.65_Na_0.35_InCl_6_ nanocrystals (NCs) via a modified hot-injection colloidal method has been reported including a photophysical study via temperature-dependent steady-state and time-resolved PL in the range of 20–300 K.

Furthermore, we address the influence of the SB and MC synthesis methods on the structural, morphological and optical properties of a set of Bi^3+^ and Na^+^-doped Cs_2_AgInCl_6_, with the general formula Cs_2_Ag_x_Na_1−x_Bi_y_In_1−y_Cl_6_, here (x = 0.4, y = 0.01, 0.02, and 0.04). To optimize Bi doping (y = 0.01 to 0.04 mol%), the Na content was fixed at x = 0.4 mol%. The resulting compositions are denoted CANBIC (SB/MC) 0.01, 0.02, and 0.04 for the sake of simplicity, representing the molar percentage of Bi^3+^. Our results reveal the importance of defect control during CANBIC synthesis and formation to achieve high PLQY.

## Experimental section

### Synthesis by SB method

 The Cs_2_Ag_0.4_Na_0.6_Bi_y_In_1−y_Cl_6_ (CANBIC), y = 0.01, 0.02, and 0.04 samples were synthesized by SB combining two transparent precursor solutions prepared in water and 2-propanol, respectively, at room temperature. Details are given in the Supplementary Information (SI).

### Synthesis by MC method

**Fig. 1:**
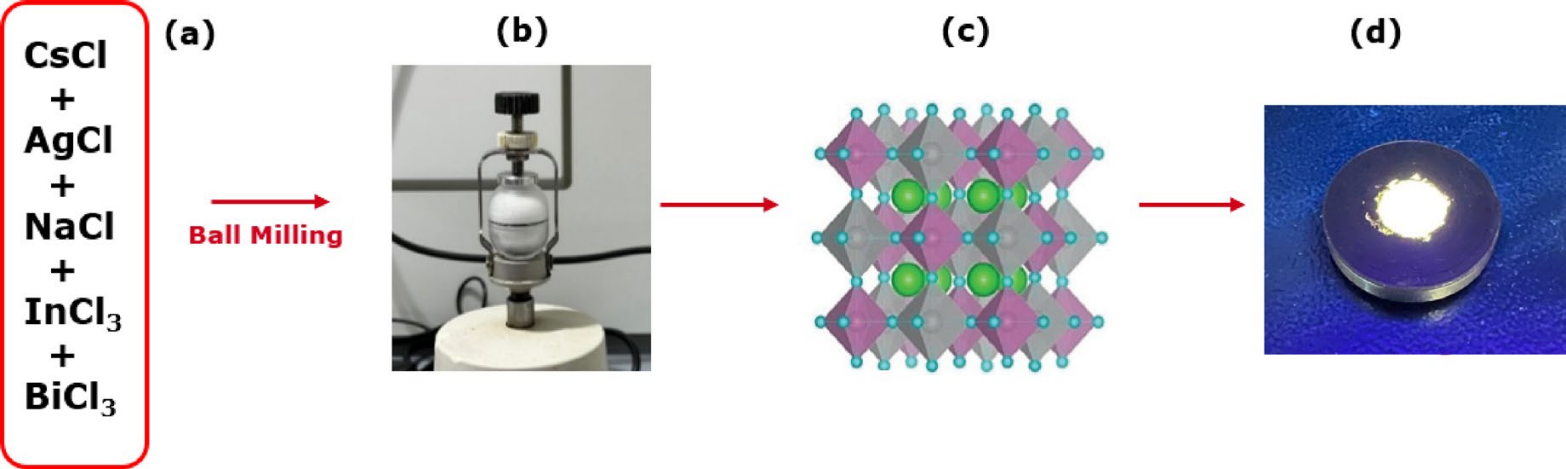
Schematic representation of the synthesis of Cs_2_Ag_0.4_Na_0.6_Bi_y_In_1−y_Cl_6_; (**a**) starting compounds; (**b**) ball milling system; (**c**) double perovskite crystal structure; and (**d**) resulting DP powder under UV light.

 The mechanochemical synthesis of the lead-free DP involved stoichiometric weighing of the solid precursor compounds CsCl, AgCl, NaCl, BiCl_3_, and InCl_3_ without additional purification, and combining them according to the scheme shown in Fig. 1:

$$2{\mathrm{CsCl}} + 0.4{\mathrm{AgCl}} + 0.6{\mathrm{NaCl}} + {\mathrm{yBiCl}}_{3} + \left( {1 - {\mathrm{y}}} \right){\text{ InCl}}_{3} \to {\mathrm{Cs}}_{2} {\mathrm{Ag}}_{{0.4}} {\mathrm{Na}}_{{0.6}} {\mathrm{Bi}}_{y} {\mathrm{In}}_{{1 - y}} {\mathrm{Cl}}_{6} .$$

The precursors were mixed in a 2:0.4:0.6:y:1-y molar ratio in a polymethyl methacrylate (PMMA) ball mill jar containing ZrO_2_ balls with a 1-cm diameter. The ball milling process was carried out at a frequency of 30 Hz with variable time intervals to achieve single-phase double perovskite compounds. The resulting powders were then heated at 100 °C to relieve the mechanical strain introduced during the milling process.

### Thermal treatment

To assess the structural stability of CANBIC samples as a function of temperature, powders from both SB and MC routes were annealed sequentially at 100, 200, 300, and 400 °C (heating rate 10 °C min^−1^, hold time 1 h at each step, Ar atmosphere). Structural changes were monitored by XRD and PL spectroscopy after each tempering step.

## Sample characterization

### Thermal, structural and microstructural characterization

 Thermogravimetric analysis (TGA) was performed on a Mettler Toledo TGA/DSC system under N_2_ atmosphere (50 mL/min) with a heating rate of 10 °C/min from 80 °C to 1100 °C. Powder X-ray diffraction analysis was conducted at room temperature to identify the crystalline phases present in the samples. The measurements were performed using a Bruker D8 Advance diffractometer operating in transmission mode. Data were collected over the 2-theta range 10° to 80°, with a step size of 0.02°, using Cu K_α1_ radiation, and with the X-ray tube operating at a voltage of 40 kV and a current of 40 mA. The crystal structure was simulated using the VESTA software with a CIF file from the ICSD database.

The microstructure was characterized by scanning electron microscopy coupled with energy-dispersive X-ray spectroscopy (SEM-EDS). SEM images were collected using the Oxford XL-30 SEM microscope, coupled with Energy Dispersive X-ray spectroscopy (EDX), and Oxford Instruments INCA energy software. Transmission electron microscopy (TEM) and selected area electron diffraction (SAED) analyses were performed utilizing a Thermo Fisher instrument operating at an accelerating voltage of 200 kV. The perovskite powders were dispersed in methanol and subsequently drop-casted onto a carbon-coated copper mesh for examination.

### Spectroscopic characterization

 Optical diffuse reflectance spectra (DRS) were acquired in a Lambda 1050 UV-Vis spectrophotometer (Perkin Elmer) equipped with an integrating sphere, using barium sulphate (BaSO_4_) as the reference standard. Photoluminescence (PL) spectra were measured in an Edinburgh FS5 spectrofluorometer with a Xe lamp as the excitation source. The absolute quantum yields of the samples were assessed by a calibrated Quantaurus spectrometer (Hamamatsu). Raman spectra measurements were conducted in a LabRAM spectrometer from Horiba Jobin Yvon using a 532 nm laser.

## Results and discussions

### High resolution transmission electron microscopy (HRTEM)

**Fig. 2:**
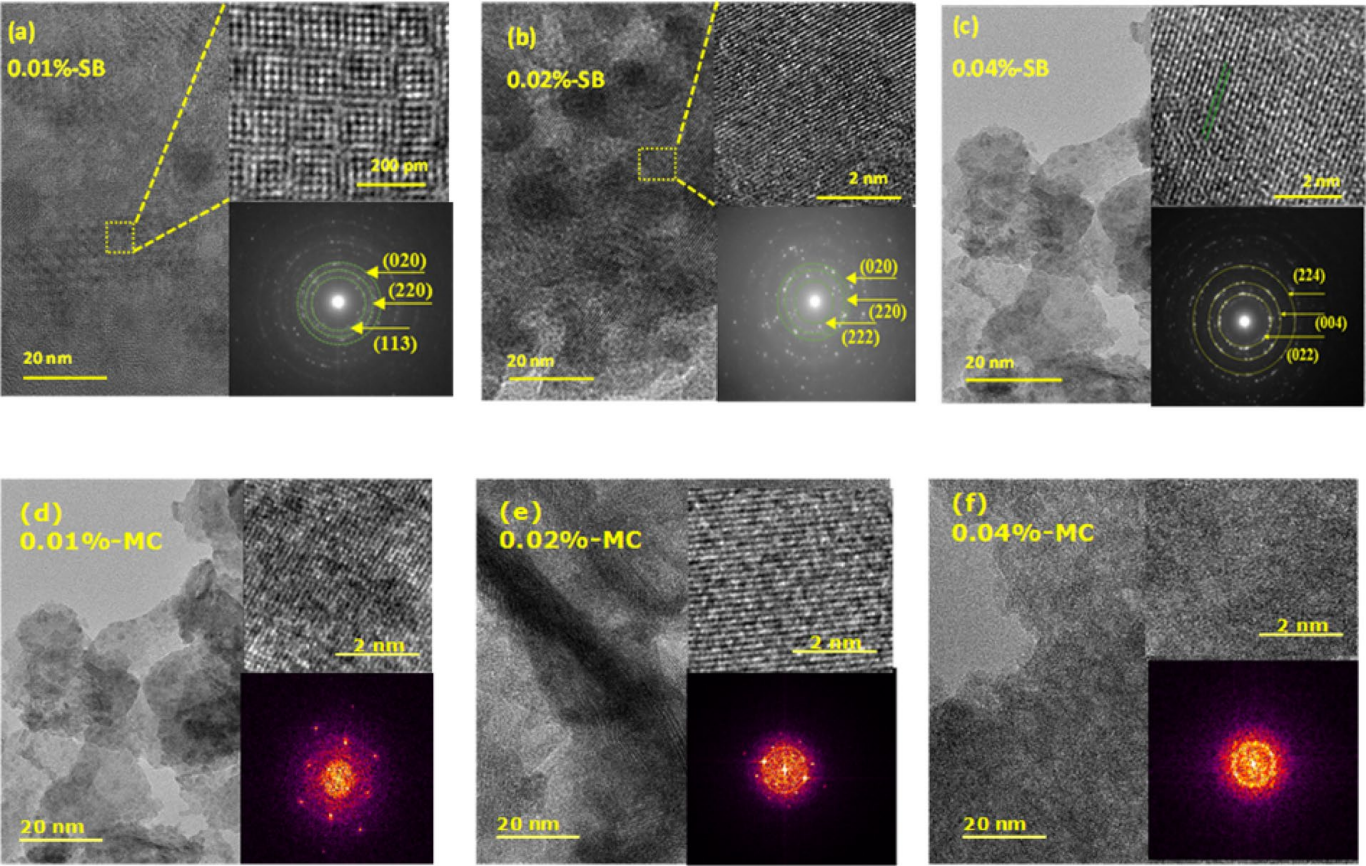
HRTEM image, and SAED pattern (inset) of (**a**) CANBIC (SB)-0.01 at%, (**b**) CANBIC (SB)-0.02 at%, (**c**) CANBIC (SB)-0.04 at%; HRTEM image and FFT pattern (inset) of (**d**) CANBIC (MC)-0.01 at%, (**e**) CANBIC (MC)-0.02 at%, (**f**) CANBIC (MC)-0.04 at%.

Figure 2(a)-(f) present the HRTEM images of the samples. For CANBIC (SB)-0.01 at% sample (Fig. 2(a)-(c)), the lattice fringes (spacing of 0.319 nm) correspond to the (113) crystal plane, which is verified in the SAED images (inset). At higher magnification, the atoms are arranged in systematic order. Upon further introduction of 0.02 at% Bi ions, an expansion in the lattice fringes (0.321 nm) corresponding to the plane (113) was observed. The observed variation in fringe spacing aligns with the shift in diffraction peaks, a phenomenon attributed to differences in Bi content that significantly influence and expand the structural symmetry. The high-resolution TEM image shows the clear lattice fringe of 0.37 nm, which corresponds to the crystal plane of the cubic Cs_2_AgInCl_6_, indicating the high crystallinity of the NCs^16^. HRTEM images (Fig. 2(d) – (f)) of CANBIC (MC) – 0.01 at%, and the corresponding Fast Fourier Transform (FFT) pattern indicate that the cubic shaped NCs possess lattice spacing around 0.36 nm corresponding to the (220) plane of Cs_2_AgInCl_6_, which further increases for CANBIC (MC)− 0.02 at% due to discrete lattice expansion. In CANBIC (MC)−0.04 at%, disorder has been observed as reflected by the halo rings in the FFT pattern.

### Crystal structure and microstructure

**Fig. 3:**
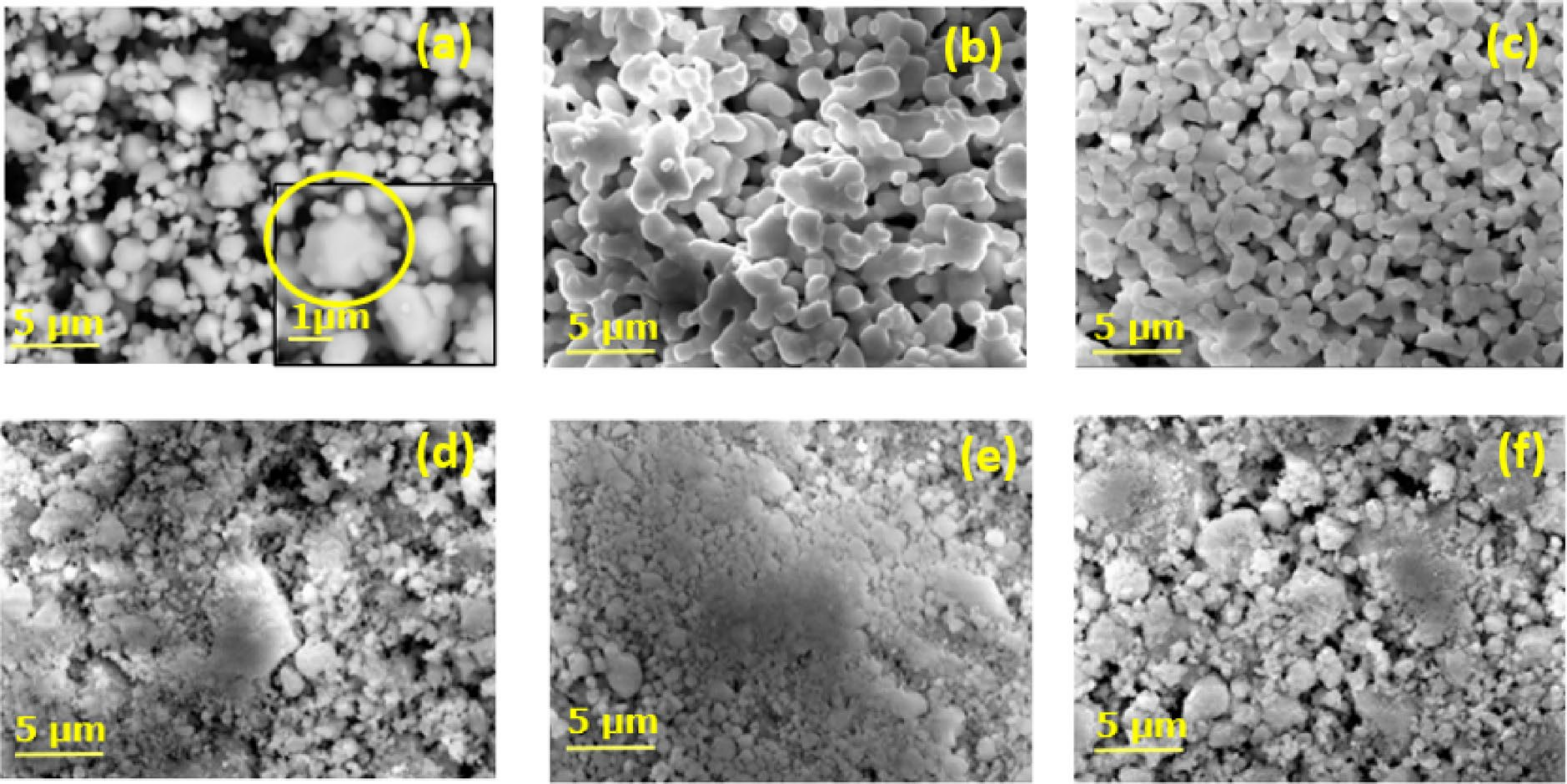
SEM images of (**a**) CANBIC (SB) -0.01% (inset showing the octahedral crystal shape), (**b**) CANBIC (SB)-0.02%, (**c**) CANBIC (SB)-0.04%; (**d**) CANBIC (MC)-0.01%, (**e**) CANBIC (MC)-0.02% and (**f**) CANBIC (MC)-0.04%. The scale bar of the SEM images is 5 μm on 10,000× magnification.

CANBIC DP samples synthesized via SB and MC resulted in white powders. SEM analysis of the CANBIC-0.01 at% sample, prepared via SB, showed a polygonal crystal structure. Incorporation of Bi^3+^ in CANBIC (SB) beyond 0.01 at% led to the growth of irregularly shaped particles as presented in Fig. 3(a)–(c). The regularity in the octahedral shape for CANBIC-0.01% is associated with symmetry and crystallinity^18^. This is attributed to the presence of lower surface energy and fewer defects. In contrast, the MC synthesis of CANBIC resulted in aggregated powder particles, making it difficult to determine the crystal morphology. This soft agglomeration is due to electrostatic attraction or van der Waals forces (refer to Fig. 3(d)–(f)).

**Fig. 4:**
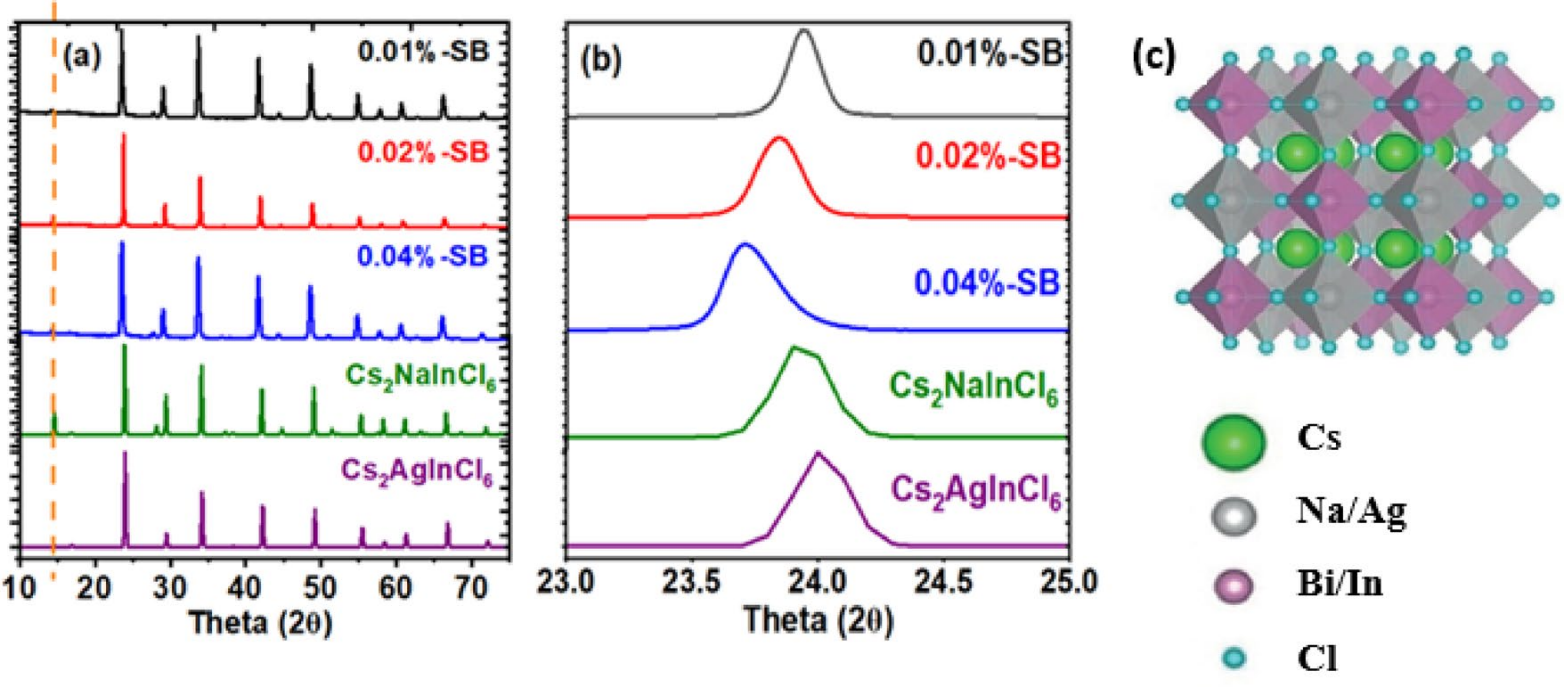
(**a**) XRD pattern of CANBIC-0.01%, 0.02%, and 0.04% synthesised using solution-based technique. (**b**) the magnified peak at 24.5° used for domain size calculation, (**c**) Structure of Cs_2_AgInCl_6_ doped with Bi and Na^17,18^.

**Fig. 5:**
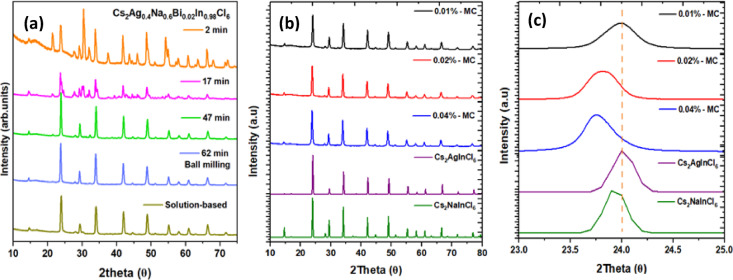
(**a**) XRD pattern of CANBIC-0.02% crystals obtained by mechanochemistry, with different milling times, (**b**) XRD of CANBIC − 0.01%, 0.02% and 0.04% synthesized by MC for 62 min of milling time, and (**c**) magnified peak at 24° which shifts towards the lower angle on introducing the Bi^3+^ ions.

To assess concentration variations of Bi content within the samples, we employed X-ray Fluorescence (XRF) (Fig. S1(a) and (b)). The spectra revealed an increase in the relative intensity of Bi L_α1_, L_β1_, and L_γ1_ signals as the concentration of Bi in CANBIC (SB) and (MC)-0.01 at%, 0.02 at%, and 0.04 at% perovskites increased. Semi-quantitative analysis of Bi L_α1_ and L_β1_ peak intensity confirms a progressive increase in Bi content from 0.01 to 0.04 at% in both SB and MC samples. For SB samples, the intensity increment between 0.02 and 0.04 at% is smaller than 0.01 and 0.02 at% which we attribute to detection sensitivity limits of XRF for trace Bi amounts in a Cl-rich matrix. The structural incorporation of Bi unambiguously confirmed by the systematic XRD peak shifts toward lower angles (Fig. 4(b) and 5(c)). The lattice parameter expansion is discussed above.

The X-ray diffraction data for the CANBIC (SB) samples with 0.01, 0.02, and 0.04 at% displayed a pattern indicative of cubic perovskite structure, conforming to the Fm-3 m space group and correlated with the reference diffraction pattern for Cs_2_AgInCl_6_ (ICSD 244519) (shown in Fig. 4(a)). In this crystal, the [AgCl_6_]^5−^ and [InCl_6_]^3−^ octahedra form a 3D framework^19^. The crystal structure remained consistent with the introduction of 0.02 at% and 0.04 at% Bi^3+^, as evidenced by the unchanged XRD patterns, presented in Fig.4(a), which indicates that Bi^3+^ replaced In^3+^ and Na^+^ replaced Ag^+^ without altering the lattice type. Upon substitution, the Bi^3+^ ions replace In^3+^, forming [BiCl_6_]^3−^ octahedra, and Na^+^ replaces Ag^+^, forming [NaCl_6_]^5−^ octahedra. The larger ionic radii of Bi^3+^ compared to In^3+^ and Na^+^ compared to Ag^+^ lead to an expansion of the crystal lattice, causing a shift of the XRD peaks towards lower angles (see Fig. 4(b))^20^. The observed lattice expansion is primarily due to the substitution of In^3+^ (ionic radius: 80 pm) by the significantly larger Bi^3+^ (103 pm). In contrast, Na^+^ (102 pm) is marginally smaller than Ag^+^ (115 pm), so Na^+^/Ag^+^ substitution does not contribute geometrically to lattice expansion; rather, it modifies the local electronic structure by disrupting Ag 4d-Cl 3p covalent hybridisation, indirectly affecting lattice parameters^20^.The crystallite size was calculated using the Scherrer formula (given in the supplementary information) and the most intense peak in the XRD pattern (Fig. 4(b)), and the results show a decrease (56.54 to 35.00 nm) with increasing.

concentration of Bi^3+^, as given in table S.1. In samples obtained via MC, the process results in particle size reduction and the continual exposure of fresh reactive surfaces, which promotes the solid-state reaction. Although the synthesis was conducted at room temperature, the local temperature during milling likely increases, favouring the reactants in overcoming the activation energy barrier for the chemical reactions^21^. Consequently, the crystallinity of the samples increased continuously with milling time. This effect was closely examined by performing a series of experiments varying the milling time from 2 to 60 min and following the corresponding changes in crystallinity via XRD (Fig. 5(a) and (b)). Below 20 min, the samples contain a mixture of different phases of cubic AgCl (ICSD 56538), Cs_2_AgInCl_6_ (a slight signature of its presence is observed in Fig. 5(a)). The crystalline structure began to develop after 47 min, achieving complete phase formation within 62 min. The diffraction peaks of all the CANBICs are well-matched with the reported structure (ICSD number 244519) of

Cs_2_AgInCl_6_. However, for the MC method, the diffraction domain size remained consistent, independent of Bi^3+^ content, with similar particle sizes observed for CANBIC samples with 0.01%, 0.02%, and 0.04 at% of Bi doping (given in Fig. 5(c), indicating the significance of milling time during the phase formation). Furthermore, a minor diffraction peak at 13.7° was evident for all samples and was attributed to the alternate arrangement of Na^+^/Ag^+^ cations within the DP structure, suggesting that Na^+^ partially occupies the atomic sites of Ag^+^ in the Cs_2_AgInCl_6_ lattice. It is generally anticipated that Ag^+^ and Na^+^ will substitute each other, given that Cs_2_AgInCl_6_ and Cs_2_NaInCl_6_ exhibit similar crystal structures and a minor lattice mismatch of 0.6%^22^. Conversely, the absence of residual peaks suggests that Na^+^ and Bi^3+^ doping effectively suppresses the formation of impurity phases. The ionic radii difference of the cations can result in symmetry breaking in the [InCl_6_]^3−^ and [AgCl_6_]^5−^ octahedra, generating localized strain within the lattice^23^.

### Raman spectroscopy

Raman spectra of CANBIC-SB and CANBIC-MC samples exhibit phonon modes at approximately 111, 140, 172, 239, and 296 ± 0.5 cm^− 1^ (Fig. S2). These are assigned as follows: the mode at 111 cm^−1^ corresponds to a metal-halide lattice translation mode; those at 140 ± 0.5 and 172 ± 0.5 cm^−1 ^are attributed to external lattice modes involving Cs^+^ and internal vibrations of [AgCl_6_]^5−^ octahedra, respectively; the band at 239 ± 0.5 cm^−1^ is the symmetric stretching mode of [InCl_6_]^3−^/[BiCl_6_]^3−^ octahedra, sensitive to local distortions upon Bi^3+^ incorporation; and the feature at 296 ± 0.5 cm^−1^ corresponds to higher-frequency metal-chloride stretching. The progressive intensity increase of the 111 ± 0.5 cm^− 1^ mode with Bi^3+^ content is consistent with either the activation of a Bi-related local mode or enhanced electron-phonon coupling arising from the greater polarizability of Bi^3+^ relative to In^3+^^24,25^. Critically, the peak positions and relative intensities are indistinguishable between SB and MC samples, confirming that both routes produce structurally equivalent double perovskite lattices at the local scale, and that the observed differences in optical performance arise from morphological and defect-related differences rather than bulk structural distinctions.

### Thermogravimetric analysis (TGA)

TGA of the synthesized CANBIC (SB) and (MC)-0.01, 0.02 and 0.04 at%, revealed no significant decomposition up to approximately 579 ± 1 °C and 586 ± 1 °C in CANBIC (SB) and CANBIC (MC), respectively (Fig. S3(a) and (b)). TGA shows two well-defined mass-loss events, occurring at ~ 580 ± 1 °C and ~ 756 ± 1 °C, and ~ 586 ± 1 °C and ~ 811 ± 1 °C for CANBIC (SB) and CANBIC (MC), respectively. The lower temperature mass loss (~ 580–586 ± 1 °C) is assigned to the initial breakdown of the double-perovskite framework with the release of volatile halogen species and partial formation of metal-chloride phases. The high-temperature mass loss (~ 756–811 ± 1 °C) corresponds to further decomposition and conversion to more stable inorganic residues as metal chlorides/oxides, indicating complete collapse of the perovskite structure^26,1^. The shift of the second decomposition step in MC samples (~ 55 °C higher) suggests a slightly improved thermal stability, reflecting differences in defect concentration, particle size or interparticle bonding introduced by the synthesis route.

### Thermal treatment of CANBICs

Heat treatment at 400 °C induced a phase separation in both CANBICs, as evidenced by X-ray diffraction (Fig. S4(a)–(f)). The initial single-phase pattern evolved into a superposition of two distinct patterns corresponding to Cs_2_AgInCl_6_ and Cs_2_NaInCl_6_, particularly noticeable below 20 degrees. At 300 °C and 400 °C, most XRD reflection peaks broadened and decreased in intensity, indicating a reduction in crystallite size and/or an increase in micro-strain defects due to thermal stress. The significant change in the lower angle region at 400 °C suggests a loss of the original long-range ordering and/or the formation of a new, segregated phase or structural distortion^26^. Apparently, heating induces cation redistribution, phase separation, and partial lattice collapse, resulting in increased structural disorder and the formation of overlapping crystalline domains. Consequently, the perovskite structure remains stable only up to approximately 300 °C, beyond which its structural integrity deteriorates.

### Optical properties - absorption

**Fig. 6:**
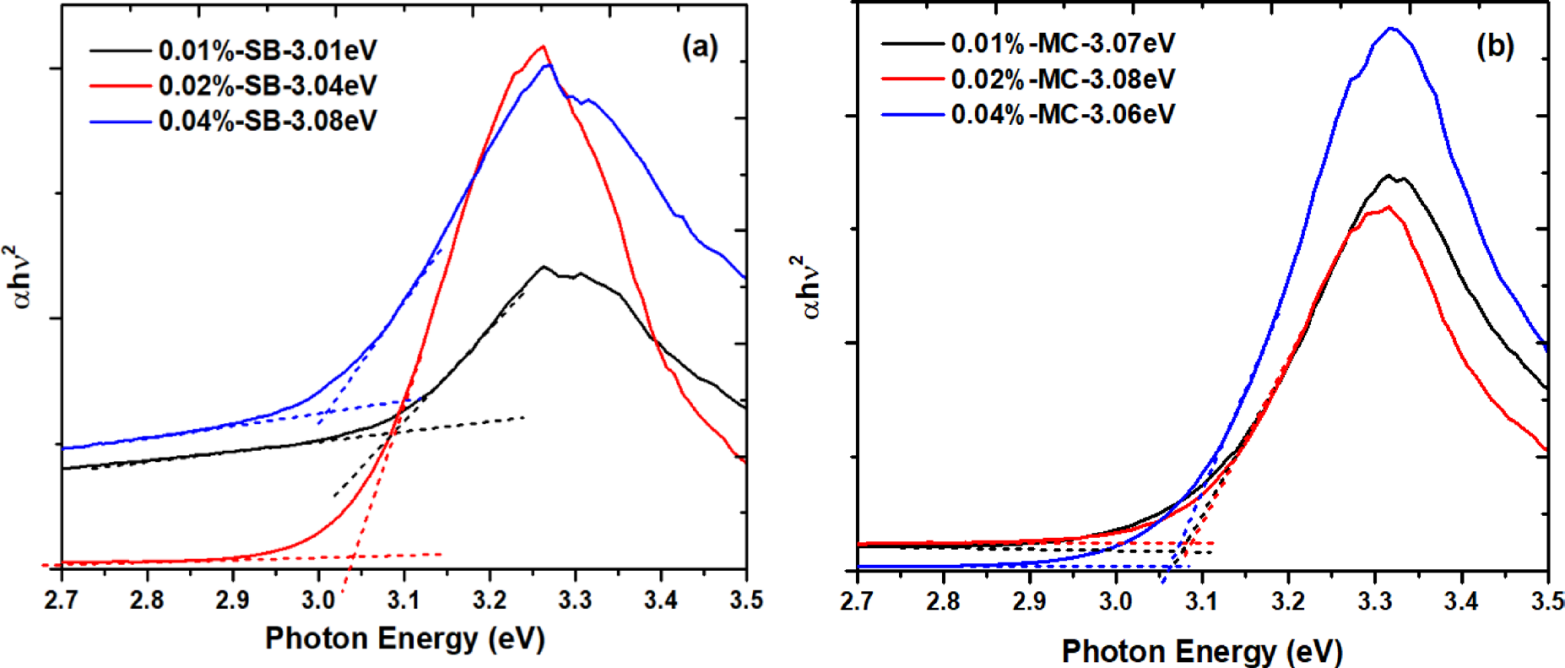
Tauc plot from UV-Vis diffuse reflectance spectra of (**a**) CANBIC (SB) −0.01%, 0.02% and 0.04%; (**b**) CANBIC (MC) –0.01%, 0.02%, and 0.04%.

The optical bandgap was evaluated from the Tauc plots, using the Kubelka-Munk^27^(formulation given in Supplementary Information) αhν^2^ vs. hν (Fig. 6(a) and (b)). We followed the protocol of Makula et al.^28^ to determine the bandgap, all CANBIC samples showed a consistent direct bandgap of ~ 3.0-3.1 ± (0.04) eV, compared to ~ 3.3 eV reported for undoped Cs_2_AgInCl_6_. The bandgap narrowing upon Bi^3+^ and Na^+^ co-incorporation arises through two distinct mechanisms: (1) Bi^3+^ introduces occupied 6s^2^ lone-pair states that hybridize with Cl-3p states, thereby shifting the VBM to higher energy and dispersing the band-edge through spin-orbit coupling; (2) Na^+^ alters the local crystal structure and modifies orbital overlap at the B-site, indirectly impacting the band-edge positions^29^. A sub-gap absorption tail extending below 3.0 ± (0.04) eV is present in all samples and is attributed to absorption by localized defect or impurity states. The broadening of the absorption feature and the presence of multiple peaks in the (3.3–4.7) ± (0.04) eV range are consistent with contribution from excitonic transitions and interband absorption.

The absorption spectra of all samples (Fig. S.5(a)–(f)) exhibit three prominent features at approximately 3.3, 3.9, and 4.6 ± 0.04 eV. The high-energy band (~ 4.6 eV) is assigned to interband transitions, primarily from Cl 3p to In 5p states of the Cs_2_AgInCl_6_ host. The lower-energy features at ~ 3.3 and ~ 3.9 eV is attributed to excitonic transitions arising from valence-band splitting induced by spin–orbit coupling and Jahn–Teller distortions coupled with Bi 6p–Cl 3p antibonding orbitals, consistent with the Elliott model for near-band-edge absorption in semiconductors^30^. The exciton features around (3.34–3.60) ± 0.04 eV broaden and intensifies with increasing Bi^3+^ content (see Fig. S6)), indicating that Bi incorporation enhances excitonic absorption while the interband absorption remains approximately constant.

### Photoluminescence emission and excitation

**Fig. 7:**
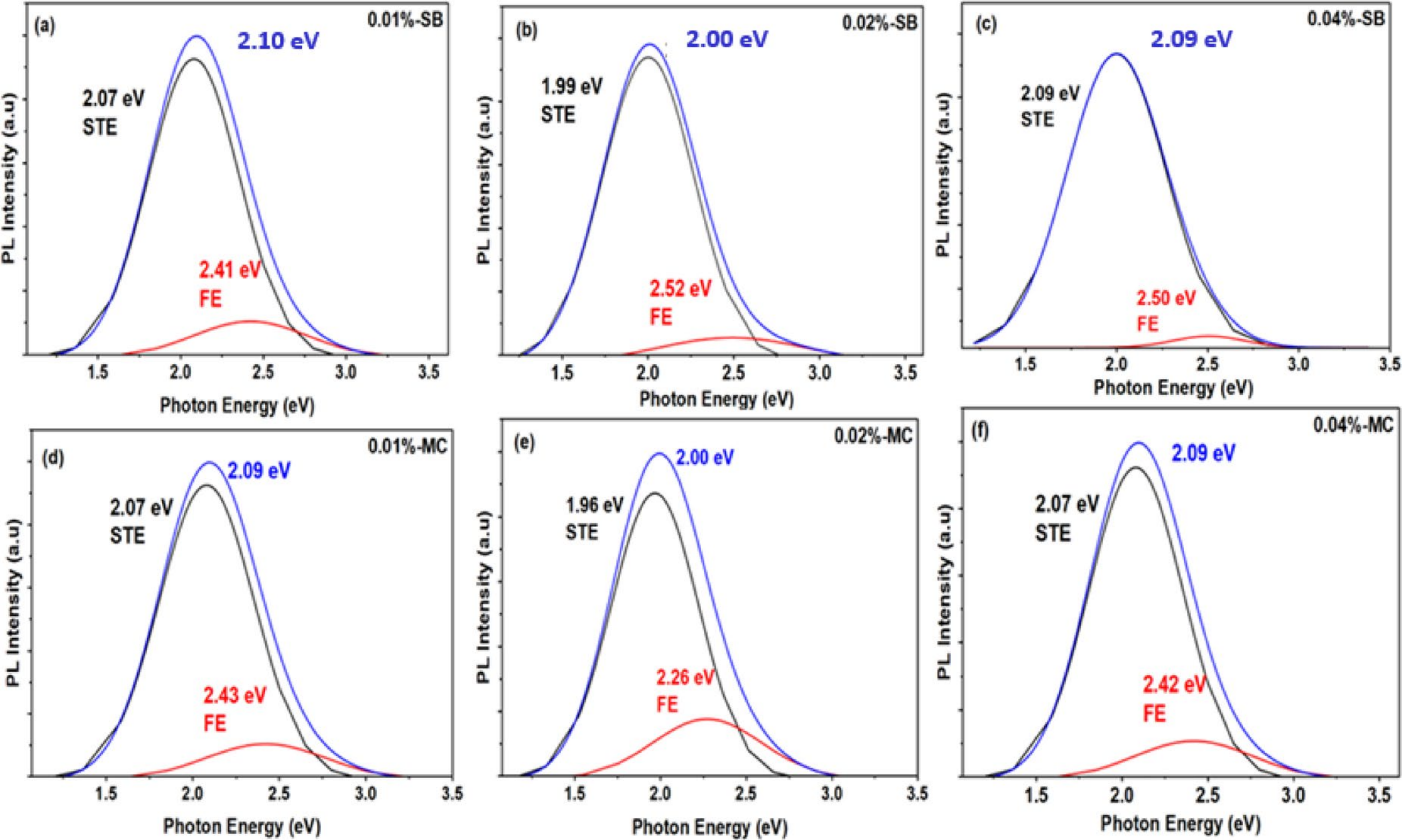
PL emission (blue line) spectra along with their Gaussian fits of (**a**) CANBIC (SB)-0.01%, (**b**) CANBIC (SB)-0.02%, (**c**) CANBIC-0.04%; (**d**) CANBIC (MC)-0.01%, (**e**) CANBIC (MC)-0.02%, (**f**) CANBIC (MC)-0.04%. (Fitted lines: Black – STE, and Red – FE)

All CANBIC samples exhibit broad, asymmetric PL emission under 345 nm (3.59 eV) excitation, with emission peaks centered in the range (2.1–2.3) ± 0.005 eV (Fig. 7(a)–(f)).

The spectra are Jacobian-corrected prior to analysis (details in the S.I.)^31,32^. The broad asymmetric (ranging between 1.37 and 3.1 eV) peak is characteristic of STE emission, arising from strong electron-phonon coupling and significant lattice relaxation in the excited state. Deconvolution of PL emission spectra was performed using a double Gaussian function, revealing two emission bands: a lower-energy component attributed to STEs (SB: 1.99–2.09 ± 0.005 eV; MC: 1.96–2.07 ± 0.005 eV and a higher-energy component attributed to free exciton (FE) recombination (SB: 2.41–2.52 eV; MC: 2.26–2.43 eV). The large Stokes shift between absorption (~ 3.3 ± 0.005 eV) and STE emission (~ 2.0 eV) is consistent with substantial lattice reorganization in the excited state, characteristic of Jahn-Teller distortion within the [AgCl_6_]^5−^ octahedra^33^. The subtle differences in the local structural and electronic environment, induced by the synthesis route, are consistent with the broader FE emission area observed in MC samples. The larger FE contribution observed in MC samples, coupled with their lower PLQY and shorter average lifetimes (see section ***Photoluminescence Quantum Yield and Decay Dynamics***), indicates a higher population of delocalized excitons that preferentially undergo non-radiative recombination rather than self-trapping. This is consistent with the elevated density of structural defects and micro-strain introduced by high-energy ball milling^34,35^ which promotes grain refinement, powder activation, and enhanced dopant homogenization, yielding the consistent EA region centered at ~ 3.54 ± 0.005 eV across all Bi concentrations and increasing non-radiative pathways relative to solution-based synthesis^22^.

**Fig. 8:**
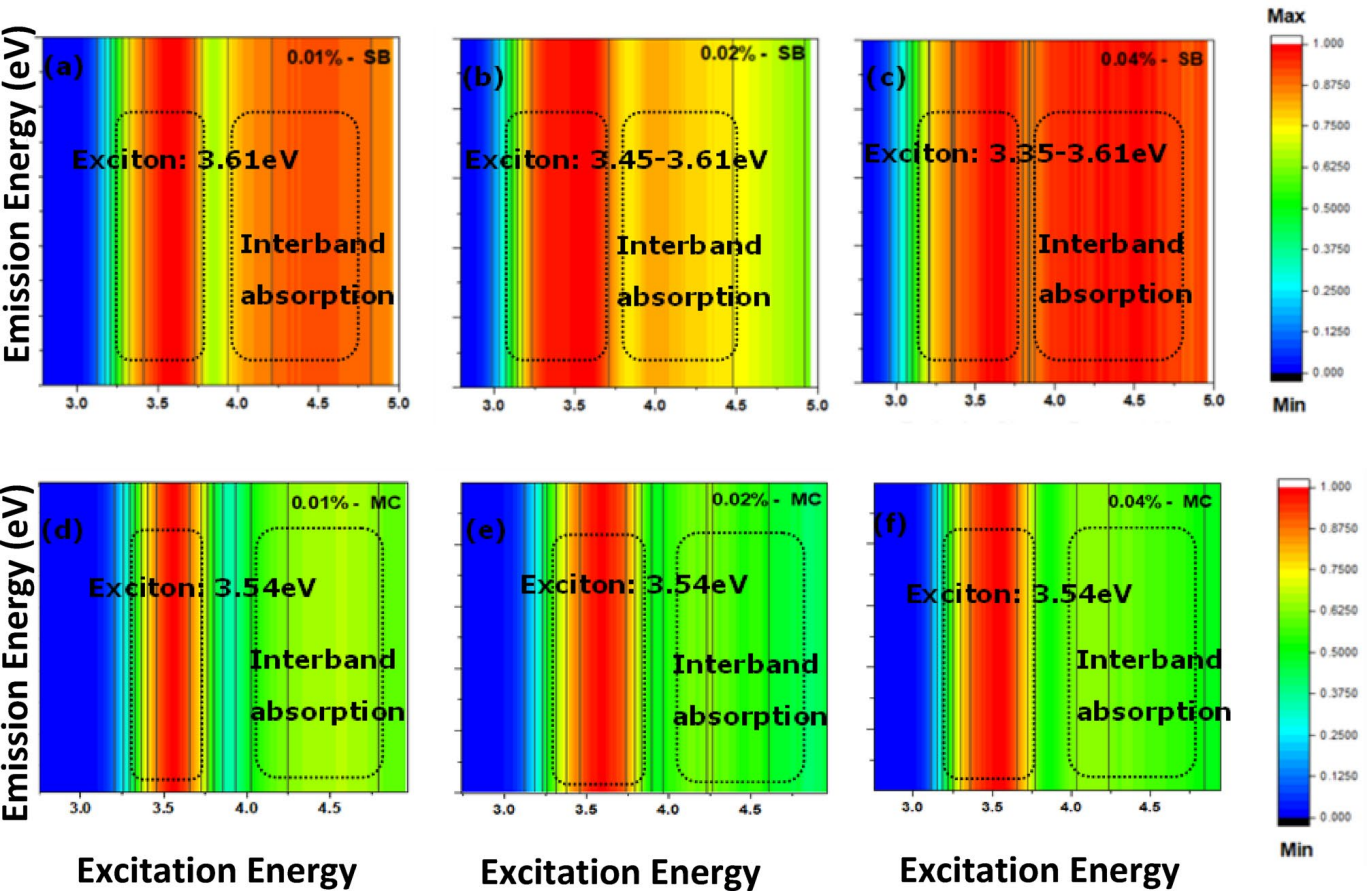
(**a**)–(**c**). 2D Photoluminescence excitation versus emission spectra of CANBIC (SB)-0.01%, 0.02% and 0.04%. (**d**)–(**f**) CANBIC (MC) −0.01%, 0.02%, and 0.04%.

2-Dimensional PLE-emission maps were obtained for all CANBIC samples (Fig. 8 (a)–(f)) illustrating a comprehensive view of excitation pathways contributing to emission spectra. With excitation spectra generated at an emission of 2.17 ± 0.005 eV, these 2D maps clearly delineate the two excitation regions: a lower-energy region corresponding to ‘exciton absorption’ and a higher-energy region corresponding to ‘interband absorption’, also illustrated in Fig. S6.

For SB samples, the EA feature appears at ~ 3.61 eV for 0.01% Bi, broadening and red-shifting progressively to (3.45–3.61) ± 0.005 eV for 0.02% Bi and (3.35–3.61) ± 0.005 eV for 0.04% Bi, indicating that increasing Bi^3+^ content modifies the excitonic binding energy and/or local electronic structure around BiCl_6_ octahedra, consistent with observed red-shifts of sub-bandgap absorption bands upon higher Bi doping in CANBICs^29,20^.

For MC samples, the EA region remains consistently centered at ~ 3.54 ± 0.005 eV across all Bi concentrations^22^, suggesting that the mechanochemical synthesis yields a more uniform local environment for exciton formation through enhanced dopant homogenization via high-energy ball milling^22^. For both synthesis methods, interband absorption begins at ~ 3.8 eV and extends to higher energies, corresponding to direct VBM and CBM transitions and the Cl 3p to In 5p transitions discussed above.

### Photoluminescence quantum yield and decay dynamics

**Fig. 9:**
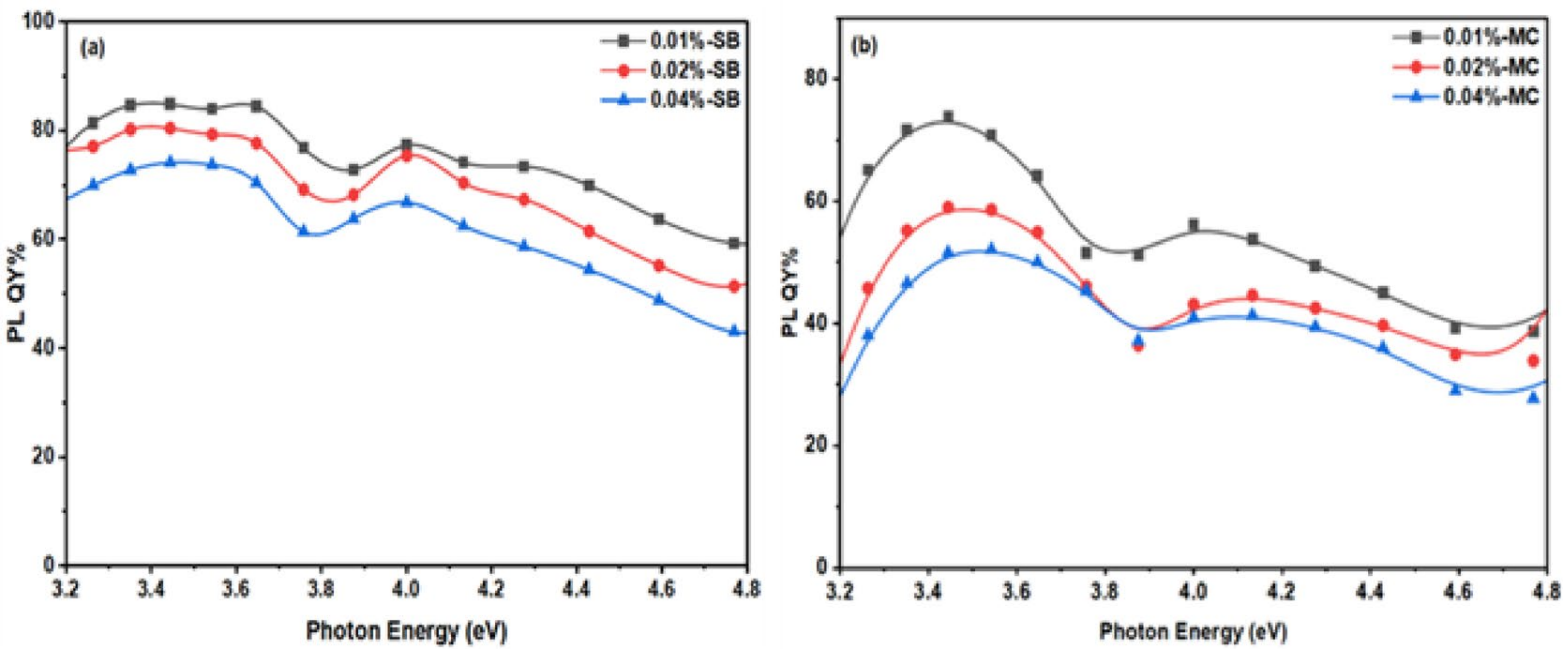
Photoluminescence quantum yield (PLQY%) at different excitation energy for: (**a**) CANBIC (SB), and (**b**) CANBIC (MC): 0.01%, 0.02%, and 0.04%.

Experimental absolute PLQY values of the two sets of CANBICs, obtained as a function of excitation energy are presented in Fig. 9 (a) and (b). The undoped Cs_2_AgInCl_6_ reference exhibits a very low PLQY of ~ 1.7%, due to the previously discussed parity-forbidden nature of the band-edge transition^36^. Upon Bi^3+^ doping, the PLQY increases and reaches a maximum of 84% at y = 0.01 for SB samples (Table 1). With a further increase in Bi^3+^ content (y = 0.02, 0.04 at%), the PLQY decreases for both SB and MC samples, due to concentration quenching – where the proximity of Bi^3+^ centers facilitates cross-relaxation and energy migration to non-radiative quenching sites. This reduced PLQY with increasing Bi content is consistent with previous studies on similar CANBIC systems. Stroyuk et al. prepared a broad range of Cs_2_Ag_x_Na_1−x_Bi_y_In_1−y_Cl_6_ compositions and reported that PLQY rises with increasing Bi concentration up to maximum of 98% at y = 0.01–0.02 at%, and then decreases with higher Bi concentrations (y < 0.01 at%)^19,20^. For Bi concentrations below y = 0.01 at%, the PLQY drops significantly, below 40% at y = 0.005 at%, due to an insufficient density of Bi^3+^ dopant centres to sustain efficient STE-mediated broadband emission across the host Cs_2_AgInCl_6_ lattice. A similar trend was observed by Stroyuk et al., where compositions with y < 0.01 at% exhibited markedly reduced radiative recombination rates. These findings from the same CANBIC family suggest that y = 0.01 at% represents a lower threshold for efficient luminescence, below which the dopant density is too small to activate the STE emission mechanism effectively. Our initial choice of y = 0.01 at% as the lowest doping level was inspired by and consistent with this established compositional boundary in the literature^11,19,20^. The relative standard deviation for PLQY measurements in the range of the observed PLQY values is 2%; uncertainties of PLQY measurements, including instrument calibration are approximately 5%^16^.

SB samples consistently exhibit higher PLQY than MC samples at equivalent Bi^3+^ content (Table 1). CANBIC (SB) − 0.01, 0.02, and 0.04 at% samples consistently exhibit higher PLQY values, ranging from 84% to 76%, as the Bi^3+^ ion content increases. Conversely, the CANBIC (MC)-0.01, 0.02, and 0.04% samples present lower PLQY values ranging from 77 to 65%. These results suggest that the MC syntheses introduce non-radiative pathways, most likely due to enhanced defects, strains, heterogeneous dopant distribution and larger surface areas associated with smaller crystallites shown in XRD and SEM images. SB synthesis allows superior morphological control afforded by, resulting in more uniform dopant distribution, lower surface defect density (consistent with the more regular crystallite morphology observed by SEM), and reduced grain boundary trapping^21,37^. The trend in PLQY with Bi^3+^ content also confirms the trend in excited-state lifetime.

**Table 1:** Evaluated values of average lifetime with standard deviation, PLQY, radiative and non-radiative rates of CANBIC synthesized by solution-based (SB) and Mechanochemistry (MC).

Compositions	Solution based		Mechanochemistry	
	τ_avg_ (µs)	PLQY %	τ_avg_ (µs)	PLQY %
CANBIC-0.01	32.7 ± 0.25	84 ± 2	30.5 ± 0.30	78 ± 2
CANBIC-0.02	27.3 ± 0.20	80 ± 2	26.9 ± 0.15	58 ± 2
CANBIC-0.04	23.4 ± 0.15	76 ± 2	23.2 ± 0.15	50 ± 2

Errors represent standard deviation across measurement repeated 3 times for each sample.

This analysis provides insight into recombination dynamics and the influence of synthesis on the photophysical behaviour of CANBIC (SB) and (MC) perovskites.

The calculated average lifetime for the CANBIC samples (SB and MC) from the time-resolved measurements (details in SI), and their PLQY values are given in Table 1.

A key observation across both plots shown in Fig. S8. (a) and (b) is the general trend of a decreasing luminescence lifetime with increasing Bi concentration. This luminescence quenching is ascribed to an increase in the non-radiative energy transfer or intermolecular interactions that dissipate energy. Comparing the two syntheses, SB exhibits slightly longer lifetimes than MC at each concentration. For instance, at 0.01 at%, SB shows a lifetime of 32.7 ± 0.25 µs (SB) and 30.5 ± 0.30 µs (MC). The reasons for the enhanced lifetime of SB include reduced structural imperfections that could act as energy traps^36^.

### Effect of tempering on photoluminescence

The influence of tempering, i.e., thermal treatment, on PL emission was investigated for all CANBIC-SB and CANBIC-MC samples over the range of RT–400 ± 1 °C (Fig. S7(a) and (b)). A non-monotonic PL intensity trend is observed: for most samples, particularly at lower Bi^3+^ concentrations, PL intensity initially increases upon tempering at 100–200 ± 1 °C. This thermally activated enhancement is attributed to defect healing — specifically, recrystallization and removal of shallow trap states (e.g., halide vacancies) that act as non-radiative recombination centres^37,29^. For MC samples, the initial PL increase also likely reflects partial relief of mechanical strain introduced during milling, improving grain boundary connectivity and reducing non-radiative pathways. Beyond 200 ± 1 °C, PL intensity decreases monotonically and nearly vanishes at 400 ± 1 °C, particularly for MC samples. This is consistent with the onset of halide volatilization and cation migration at elevated temperatures, which generate non-radiative recombination centres^38^. The concurrent XRD evidence for phase separation at s400 ± 1 °C confirms that the loss of PL arises from structural degradation rather than simple thermal quenching. A systematic red shift of the PL band with increasing tempering temperature is observed for all samples, attributed to lattice thermal expansion and enhanced electron-phonon coupling, which deepen the STE potential well and reduce the emitted photon energy^38^.

## Conclusion

In this work, we successfully employed both solution-based and mechanochemical methods to synthesize highly luminescent lead-free double perovskites with the general formula Cs₂Ag₀.₄Na₀.₆BiyIn₁₋yCl₆ (y = 0.01, 0.02, and 0.04 mol%), exhibiting a cubic structure. The incorporation of Bi^3+^ and Na^+^ ions led to distortion of the crystal lattice, modified electronic structure and allowed optical transitions due to the different ionic radii of the dopants. As a consequence of reduced symmetry, allowed optical transitions and induced STEs, radiative recombination is enhanced, thus increasing PL intensity, while thermal studies demonstrated stability up to 300 °C for luminescent properties, with decomposition of the compounds occurring above 560 °C. Comparative analysis revealed significant differences in the materials characteristics based on the SB or MC synthesis techniques. The optimal doping concentration (y = 0.01) resulted in the highest photoluminescence quantum yield of up to 84% in SB samples. These samples consistently exhibited a superior optical performance, characterized by enhanced radiative rates and improved defect passivation, attributed to better control over particle morphology and dopant distribution. The mechanochemical synthesis, offers a rapid and green solvent-free approach, also producing highly luminescent perovskites, but it led to aggregation of particles and enhanced the density of defects. This led to lower PLQYs and higher susceptibility to thermal degradation. Time-resolved photoluminescence revealed biexponential decay kinetics, suggesting the presence of multiple recombination pathways introduced by both preparation methods, but with varying contributions to radiative and non-radiative processes depending on the synthesis route. Precise control of Na^+^ and Bi^3+^ doping, particularly through solution-based methods, enables effective tuning of the materials’ structural, electronic, and optical properties, positioning CANBIC materials as promising candidates for next-generation sustainable white light-emitting diodes and advanced optoelectronic devices. The bandgap narrowing and PLQY enhancement observed in the CANBIC samples is ascribed to the theoretical findings reported for this material class which was briefly summarized in the introduction. The presented work advances beyond a closely related study by Huang et al.^15^ reporting the synthesis of Bi^3+^-doped Cs_2_Ag_0.65_Na_0.35_InCl_6_ nanocrystals (NCs) via a modified hot-injection colloidal method by (1) achieving significantly higher PLQY (84%) through significantly lower Bi doping (0.01–0.04 at%); (2) providing the first systematic comparison of two environment friendly synthesis routes (SB and MC) and (3) demonstrating superior thermal stability up to 300 °C. These properties are critical for solid-state lighting applications.

## Supplementary Information

Below is the link to the electronic supplementary material.

Supplementary Material 1

## Data Availability

The datasets used and/or analyzed during the current study are available from the corresponding author on reasonable request.
