# Supplementary material for: Comparing the structure and photoluminescence properties of Bi-doped lead-free double perovskites prepared by solution-based synthesis and green mechanochemistry
Source: Sci Rep. 2026 Aug 10;16:24703. doi: 10.1038/s41598-026-66090-3 (PMC13458326; doi:10.1038/s41598-026-66090-3)
Supplement: Supplementary file 1 [file 41598_2026_66090_MOESM1_ESM.docx]

**Supplementary Information**

**Comparing the Structure Analytical and Luminescence Properties of Bi-doped Lead-Free Double Perovskites Prepared by Solution-Based Synthesis and Green Mechanochemistry**

Shweta Chahal^1^, Christian Würth^1^, Arne Güttler^1^, Ute Resch-Genger^1^, Andrea Simone Stucchi de Camargo^1,2^

^1^*Federal Institute for Material Research and Testing (BAM), Berlin, Germany – 12489*

*^2^ Otto-Schott Institute of Materials Research (OSIM), Friedrich-Schiller University, Jena 07743, Germany.*

1. **Materials and Methods**

***Synthesis by SB method.*** The Cs_2_Ag_0.4_Na_0.6_Bi_y_In_1-y_Cl_6_ (CANBIC), y=0.01,0.02, and 0.04 at.% samples were synthesized by SB combining two transparent precursor solutions prepared in water and 2-propanol, respectively, under ambient room temperature.

A stock BiCl_3_ solution (1.0 M) was first prepared in 4.0 M HCl in which BiCl_3_ completely dissolved without forming precipitate due to hydrolysis. Similarly, a stock of InCl_3_ solution (1.0 M) in 4.0 M HCl was prepared. The separate stock solution of silver nitrate, sodium acetate and cesium acetate were prepared in deionized water.

For synthesis of perovskites two precursor solutions were prepared separately as described below and then precursor 1 was mixed with precursor 2 under vigorous stirring.

Precursor 1 was prepared by adding 2 mL of a 1.0 M stock BiCl_3_ solution, 98 mL of a 1.0 M stock InCl_3_ solution, 0.10 mL of concentrated 37 wt.% HCl, and 5 mL of 2-propanol to a beaker. To avoid HCl evaporation, the acid was mixed with the 2-propanol separately prior to being added as a single reactant.

Precursor 2 was formulated by mixing 5 mL of 2-propanol, 1 mL of deionized water, 0.4 mL of a 1.0 M AgNO_3_ solution, and 0.25 mL of a 5.0 M aqueous ammonia solution in a beaker, under constant agitation. Ammonia was added to facilitate the formation of Ag(I)-NH_3_ upon subsequent addition of 0.025 mL of sodium acetate solution (4.0M) and 0.06 mL cesium acetate solution (4.0 M) under vigorous stirring.

Precursor 1 was then combined with precursor 2 in the appropriate proportions according to composition, yielding a white precipitate. This precipitate was maintained in the supernatant for 24 hours to facilitate crystallization. The precipitate was then separated and purified by centrifugation with 2-propanol. The precipitate was dried at 60 °C under ambient conditions for subsequent characterization ^28.^

**Scherrer Formula for crystalline size calculation**

d=kλ/βcosθ, where d is the crystalline size, λ is the wavelength of x-ray radiation, k = 0.94 (for cubic crystal) is a constant depending on the shape factor, and β is the full width at the half maximum (in radian)

**Kubelka Munk Formulation**

F(R) = (1−R)²/2R = α/S; R is reflectance, F(R) is proportional to α which is absorption coefficient, S is scattering coefficient

(αhν)^n^ = B(hν−Eg); n = 2 for direct bandgap

Diffuse reflectance spectra were collected using a UV-Vis spectrometer equipped with an integrating sphere, with BaSO_4_ as the white reference standard. Optical bandgap energies are determined from Tauc plots of (αhν)^2^ Vs hν. To account for the subjectivity in the linear fitting and intersection with baseline, each Tauc plot evaluated three times and the bandgap is reported as the mean ± standard deviation of these fits.

**Calculation of Average lifetime**

 τ_avg_ = (B_1_τ_1_^2^ + B_2_τ_2_^2^) / (B_1_τ_1_ + B_2_τ_2_)

B_1_ and B_2_ are constants, and τ_1_ and τ_2_ correspond to shorter and longer lifetime components, respectively. All TRPL decays are fitted to a biexponential model. The standard error for each sample was determined by repeating the measurement in triplicate (intra-batch). The mean and standard deviation across these repeated measurements is reported.

**Jacobian Conversion for Photoluminescence Emission**

PL spectra are generally measured as function of (I(λ), indicating number of photons per second detected within a specific wavelength interval dλ. Therefore, Gaussian fitting is done on ‘per-energy-interval’ basis to analyse the population of emitters. As dλ and dE are not linearly related (E α 1/λ), the conversion requires a Jacobian determinant (dλ/dE) to transform the density of states correctly.

**The Transformation Formula**

To convert a PL spectrum from the I(λ) to I(E) intensity must be scaled as follows:

I(E) = I(λ) . |d I(λ)/dE|

Since E = hc / λ, the conversion factor is proportional to λ^2^ (or 1/E^2^)):

I(E) = I(λ). λ^2^ / hc

The uncertainty in the photoluminescence (PL) emission and excitation measurements is primarily governed by the instrumental precision. Based on the instrumental calibration and specifications, the uncertainty in energy is estimated to be within ± 0.005 eV, while the relative intensity uncertainty is estimated to be within 5%.

**Table S.1.** Calculated domain size (in nm) using Scherrer formula for Solution based and Mechanochemically synthesized CANBICs

**Results and Disucussions**

**Fig.S2.** (a) Raman spectra of CANBIC (SB) – 0.01, 0.02, and 0.04%; (b) CANBIC (MC) – 0.01, 0.02, and 0.04%.

**Fig.S1.** X-Ray Reflective Fluorescence (a). CANBIC (SB) -0.01, 0.02, 0.04%; (b) CANBIC (MC)-0.01, 0.02, 0.04%.

**Fig.S4.** X-Ray diffraction of **(**a) – (c). CANBIC (SB) -0.01, 0.02, 0.04%; (d)-(f). CANBIC

(MC)-0.01, 0.02, 0.04% at different temperature from room temperature to 400°C with the

interval of 100°C.

**Fig.S4.** X-Ray diffraction of **(a) – (c).** CANBIC (SB) -0.01, 0.02, 0.04%; **(d)-(f)**. CANBIC (MC)-0.01, 0.02, 0.04% at different temperature from room temperature to 400°C with the interval of 100°C.

**Fig.S3.** Thermogravimetric analysis curve of (a). CANBIC (SB) -0.01, 0.02, 0.04%; (b) CANBIC (MC)-0.01, 0.02, 0.04%.

**Fig.S5.** Absorbance spectra of (a) CANBIC (SB) **-**0.01%, (b) CANBIC (SB) – 0.02%, (c) CANBIC – 0.04%; (d) CANBIC (MC)-0.01%, (e) CANBIC (MC)-0.02%, (f) CANBIC (MC)-0.04%.

**Fig.S6.** Excitation spectra of (a) CANBIC (SB) -0.01, 0.02, 0.04% and (b) CANBIC (MC)-0.01, 0.02, 0.04%.

**Fig.S7.** PL emission intensity of thermally treated CANBIC from room temperature to 400°C with the interval of 100°C under inert environment: (a) CANBIC (SB)-0.01%, (b) CANBIC (SB)-0.02%, (c) CANBIC (SB)-0.04%; (d) CANBIC (MC)-0.01%, (e) CANBIC (MC)-0.02%, (f) CANBIC (MC)-0.04%.

**Fig.S8.** PL decay curves for (a) CANBIC (SB) – 0.01, 0.02, 0.04% and (b) CANBIC (MC) – 0.01, 0.02, and 0.04%, under the 365 nm excitation and emission wavelength at 560 nm (CANBIC (SB) and 570 nm (CANBIC (MC).
